# Supplementary material for: Nasopharyngeal Carcinoma Radiomic Evaluation with Serial PET/CT: Exploring Features Predictive of Survival in Patients with Long-Term Follow-Up
Source: Cancers (Basel). 2022 Jun 24;14(13):3105. doi: 10.3390/cancers14133105 (PMC9264840; doi:10.3390/cancers14133105)
Supplement: Supplementary file 1 [file cancers-14-03105-s001.zip › cancers-1744543-supplementary.pdf]

Supplementary Table. List of all statistically significant features ($p<0.01$) from the time-dependent model

CT + PET 40% for overall survival

Radiomic features	HR (95% CI)	p-value
CT_CONVENTIONAL_TLG.mL..onlyForPETorNM.	1.92 (1.33,2.77)	0.0004
CT_DISCRETIZED_TLG.mL..onlyForPETorNM.	1.93 (1.34,2.79)	0.0004
CT_SHAPE_Volume.mL.	1.93 (1.34,2.79)	0.0004
CT_SHAPE_Volume.vx.	1.93 (1.34,2.79)	0.0004
CT_SHAPE_Surface.mm2..onlyFor3DROI.	2.02 (1.36,3)	0.0005
CT_GLRLM_GLNU	1.99 (1.35,2.94)	0.0005
CT_GLRLM_RLNU	1.9 (1.32,2.73)	0.0006
CT_NGLDM_Busyness	2.54 (1.29,5)	0.0069
CT_GLZLM_LZE	2.33 (1.44,3.78)	0.0006
CT_GLZLM_LZLGE	2.34 (1.44,3.79)	0.0006
CT_GLZLM_LZHGE	2.32 (1.43,3.76)	0.0006
CT_GLZLM_GLNU	1.93 (1.32,2.83)	0.0008
CT_GLZLM_ZLNU	1.74 (1.26,2.41)	0.0007
PET_CONVENTIONAL_SUVbwmin	3.26 (1.61,6.59)	0.0010
PET_CONVENTIONAL_SUVbwmean	2.94 (1.56,5.54)	0.0008
PET_CONVENTIONAL_SUVbwstd	2.88 (1.6,5.17)	0.0004
PET_CONVENTIONAL_SUVbwmax	2.66 (1.56,4.55)	0.0004
PET_CONVENTIONAL_SUVbwQ1	3.13 (1.58,6.22)	0.0011
PET_CONVENTIONAL_SUVbwQ2	2.78 (1.49,5.17)	0.0013
PET_CONVENTIONAL_SUVbwQ3	2.83 (1.54,5.21)	0.0008
PET_CONVENTIONAL_SUVbwpeakSphere0.5mL.value. only.for.PET.or.NM.	2.55 (1.48,4.42)	0.0008
PET_CONVENTIONAL_TLG.mL..onlyForPETorNM.	1.61 (1.22,2.11)	0.0007
PET_DISCRETIZED_SUVbwmin	3.94 (1.75,8.88)	0.0009
PET_DISCRETIZED_SUVbwmean	3.33 (1.63,6.78)	0.0009
PET_DISCRETIZED_SUVbwstd	2.8 (1.49,5.26)	0.0014
PET_DISCRETIZED_SUVbwmax	2.79 (1.55,5.02)	0.0006
PET_DISCRETIZED_SUVbwQ1	3.68 (1.69,8.01)	0.0010
PET_DISCRETIZED_SUVbwQ2	3.32 (1.63,6.78)	0.0009
PET_DISCRETIZED_SUVbwQ3	3.2 (1.58,6.46)	0.0012
PET_DISCRETIZED_SUVbwpeakSphere0.5mL.value. only.for.PET.or.NM.	2.56 (1.48,4.45)	0.0008
PET_DISCRETIZED_TLG.mL..onlyForPETorNM.	1.6 (1.22,2.1)	0.0008
PET_GLCM_Contrast..Variance.	7.63 (2.3,25.28)	0.0009

PET_GLCM_Dissimilarity	4.81 (1.77,13.09)	0.0021
PET_GLRLM_HGRE	2.93 (1.54,5.58)	0.0011
PET_GLRLM_SRHGE	2.82 (1.52,5.26)	0.0011
PET_GLRLM_LRHGE	6.52 (2.12,20.07)	0.0011
PET_GLZLM_HGZE	2.9 (1.52,5.54)	0.0012
PET_GLZLM_SZHGE	2.98 (1.55,5.74)	0.0011
PET_GLZLM_GLNU	2.26 (1.46,3.49)	0.0002
PET_GLZLM_ZLNU	2.1 (1.39,3.16)	0.0004
PET_GLZLM_ZP	4.83 (1.65,14.17)	0.0041

CT + PET 70% for overall survival

**Radiomic features**	**HR (95% CI)**	**p-value**
CT_CONVENTIONAL_TLG.mL..onlyForPETorNM.	1.93 (1.34,2.77)	0.0004
CT_DISCRETIZED_TLG.mL..onlyForPETorNM.	1.94 (1.34,2.8)	0.0004
CT_SHAPE_Volume.mL.	1.94 (1.34,2.8)	0.0004
CT_SHAPE_Volume.vx.	1.94 (1.34,2.8)	0.0004
CT_SHAPE_Surface.mm2..onlyFor3DROI.	2.03 (1.37,3.02)	0.0004
CT_GLRLM_GLNU	2 (1.35,2.95)	0.0005
CT_GLRLM_RLNU	1.9 (1.32,2.74)	0.0006
CT_NGLDM_Busyness	2.61 (1.33,5.11)	0.0053
CT_GLZLM_LZE	2.34 (1.44,3.79)	0.0006
CT_GLZLM_LZLGE	2.34 (1.44,3.81)	0.0006
CT_GLZLM_LZHGE	2.33 (1.44,3.78)	0.0006
CT_GLZLM_GLNU	1.93 (1.32,2.83)	0.0007
CT_GLZLM_ZLNU	1.75 (1.27,2.41)	0.0007
PET_CONVENTIONAL_SUVbwmin	2.22 (1.43,3.42)	0.0003
PET_CONVENTIONAL_SUVbwmean	2.37 (1.47,3.81)	0.0004
PET_CONVENTIONAL_SUVbwstd	4.51 (1.85,11)	0.0009
PET_CONVENTIONAL_SUVbwmax	2.64 (1.55,4.51)	0.0004
PET_CONVENTIONAL_SUVbwQ1	2.39 (1.49,3.84)	0.0003
PET_CONVENTIONAL_SUVbwQ2	2.35 (1.46,3.78)	0.0004
PET_CONVENTIONAL_SUVbwQ3	2.38 (1.47,3.87)	0.0004
PET_CONVENTIONAL_TLG.mL..onlyForPETorNM.	19.85 (3.35,117.53)	0.0010
PET_DISCRETIZED_SUVbwmin	2.21 (1.43,3.42)	0.0004
PET_DISCRETIZED_SUVbwmean	2.35 (1.46,3.76)	0.0004
PET_DISCRETIZED_SUVbwstd	4.74 (1.92,11.67)	0.0007
PET_DISCRETIZED_SUVbwmax	2.74 (1.58,4.74)	0.0003
PET_DISCRETIZED_SUVbwQ1	2.38 (1.48,3.84)	0.0004

PET_DISCRETIZED_SUVbwQ2	2.36 (1.47,3.78)	0.0004
PET_DISCRETIZED_SUVbwQ3	2.35 (1.46,3.79)	0.0005
PET_DISCRETIZED_TLG.mL..onlyForPETorNM.	19.58 (3.26,117.37)	0.0011
PET_DISCRETIZED_HISTO_Entropy_log10	3.16 (1.35,7.43)	0.0082
PET_DISCRETIZED_HISTO_Entropy_log2	3.16 (1.35,7.43)	0.0082

CT + PET 40% for progression-free survival

**Radiomic features**	**HR (95% CI)**	**p-value**
CT_CONVENTIONAL_TLG.mL..onlyForPETorNM.	1.51 (1.12,2.03)	0.0067
CT_DISCRETIZED_TLG.mL..onlyForPETorNM.	1.51 (1.12,2.04)	0.0065
CT_SHAPE_Volume.mL.	1.51 (1.12,2.04)	0.0065
CT_SHAPE_Volume.vx.	1.51 (1.12,2.04)	0.0065
CT_GLRLM_GLNU	1.55 (1.13,2.13)	0.0065
CT_GLRLM_RLNU	1.49 (1.1,2.02)	0.0094
CT_GLZLM_LZE	1.76 (1.17,2.64)	0.0063
CT_GLZLM_LZLGE	1.76 (1.17,2.65)	0.0063
CT_GLZLM_LZHGE	1.76 (1.17,2.64)	0.0064
CT_GLZLM_GLNU	1.53 (1.11,2.1)	0.0089
CT_GLZLM_ZLNU	1.42 (1.09,1.85)	0.0089
PET_CONVENTIONAL_SUVbwmin	2.42 (1.26,4.63)	0.0077
PET_CONVENTIONAL_SUVbwmean	2.24 (1.25,4)	0.0064
PET_CONVENTIONAL_SUVbwstd	2.13 (1.27,3.59)	0.0042
PET_CONVENTIONAL_SUVbwmax	1.98 (1.26,3.12)	0.0033
PET_CONVENTIONAL_SUVbwQ1	2.35 (1.25,4.42)	0.0077
PET_CONVENTIONAL_SUVbwQ2	2.19 (1.23,3.91)	0.0079
PET_CONVENTIONAL_SUVbwQ3	2.2 (1.25,3.86)	0.0060
PET_CONVENTIONAL_SUVbwpeakSphere0.5mL.value. only.for.PET.or.NM.	2.05 (1.28,3.3)	0.0030
PET_CONVENTIONAL_TLG.mL..onlyForPETorNM.	1.45 (1.14,1.85)	0.0027
PET_DISCRETIZED_SUVbwmin	2.85 (1.41,5.79)	0.0037
PET_DISCRETIZED_SUVbwmean	2.49 (1.34,4.65)	0.0040
PET_DISCRETIZED_SUVbwstd	2.14 (1.22,3.76)	0.0084
PET_DISCRETIZED_SUVbwmax	2.14 (1.29,3.56)	0.0033
PET_DISCRETIZED_SUVbwQ1	2.72 (1.39,5.35)	0.0036
PET_DISCRETIZED_SUVbwQ2	2.5 (1.34,4.66)	0.0039
PET_DISCRETIZED_SUVbwQ3	2.45 (1.32,4.53)	0.0043
PET_DISCRETIZED_SUVbwpeakSphere0.5mL.value. only.for.PET.or.NM.	2.06 (1.28,3.31)	0.0029

PET_DISCRETIZED_TLG.mL..onlyForPETorNM.	1.45 (1.13,1.85)	0.0030
PET_GLCM_Contrast..Variance.	4.64 (1.64,13.16)	0.0039
PET_GLRLM_HGRE	2.3 (1.31,4.03)	0.0037
PET_GLRLM_SRHGE	2.23 (1.3,3.84)	0.0038
PET_GLRLM_LRHGE	4.27 (1.6,11.39)	0.0038
PET_GLZLM_HGZE	2.29 (1.31,4.04)	0.0039
PET_GLZLM_SZHGE	2.35 (1.33,4.15)	0.0031
PET_GLZLM_GLNU	1.67 (1.23,2.26)	0.0011
PET_GLZLM_ZLNU	1.75 (1.25,2.45)	0.0012

CT + PET 70% for progression-free survival

**Radiomic features**	**HR (95% CI)**	**p-value**
CT_CONVENTIONAL_TLG.mL..onlyForPETorNM.	1.51 (1.12,2.03)	0.0064
CT_DISCRETIZED_TLG.mL..onlyForPETorNM.	1.52 (1.13,2.04)	0.0061
CT_SHAPE_Volume.mL.	1.52 (1.13,2.04)	0.0061
CT_SHAPE_Volume.vx.	1.52 (1.13,2.04)	0.0061
CT_SHAPE_Surface.mm2..onlyFor3DROI.	1.53 (1.11,2.11)	0.0086
CT_GLRLM_GLNU	1.56 (1.13,2.13)	0.0061
CT_GLRLM_RLNU	1.5 (1.11,2.02)	0.0087
CT_GLZLM_LZE	1.77 (1.18,2.65)	0.0060
CT_GLZLM_LZLGE	1.77 (1.18,2.65)	0.0059
CT_GLZLM_LZHGE	1.76 (1.18,2.64)	0.0060
CT_GLZLM_GLNU	1.53 (1.11,2.1)	0.0086
CT_GLZLM_ZLNU	1.42 (1.09,1.85)	0.0085
PET_CONVENTIONAL_SUVbwmin	1.75 (1.21,2.53)	0.0031
PET_CONVENTIONAL_SUVbwmean	1.84 (1.22,2.77)	0.0038
PET_CONVENTIONAL_SUVbwstd	2.95 (1.3,6.69)	0.0098
PET_CONVENTIONAL_SUVbwmax	1.97 (1.25,3.11)	0.0036
PET_CONVENTIONAL_SUVbwQ1	1.84 (1.23,2.76)	0.0031
PET_CONVENTIONAL_SUVbwQ2	1.83 (1.21,2.76)	0.0041
PET_CONVENTIONAL_SUVbwQ3	1.85 (1.21,2.82)	0.0044
PET_CONVENTIONAL_TLG.mL..onlyForPETorNM.	5.67 (1.75,18.39)	0.0039
PET_DISCRETIZED_SUVbwmin	1.74 (1.2,2.53)	0.0034
PET_DISCRETIZED_SUVbwmean	1.83 (1.22,2.74)	0.0037
PET_DISCRETIZED_SUVbwmax	2.02 (1.26,3.24)	0.0036
PET_DISCRETIZED_SUVbwQ1	1.84 (1.23,2.76)	0.0033
PET_DISCRETIZED_SUVbwQ2	1.83 (1.22,2.76)	0.0037

PET_DISCRETIZED_SUVbwQ3	1.83 (1.2,2.78)	0.0047
PET_DISCRETIZED_TLG.mL..onlyForPETorNM.	5.68 (1.73,18.69)	0.0042
